# Effects of heat stress on the expression of the coxsackievirus and adenovirus receptor in mouse skin keratinocytes

**DOI:** 10.3892/etm.2013.1230

**Published:** 2013-07-23

**Authors:** XIANGDONG DENG, CHIYU JIA, FUXING CHEN, JUNQUAN LIU, ZONGHAI ZHOU

**Affiliations:** 1 The Graduate School, Chinese People’s Liberation Army Medical College, Beijing 100039;; 2 Plastic Beauty and Burn Repair Center, The 309th Hospital of the Chinese PLA, Beijing 100091;; 3 The 97th Hospital of the Chinese PLA, Xuzhou, Jiangsu 221004, P.R. China

**Keywords:** heat stress, keratinocytes, coxsackie-adenovirus receptor, burn wound

## Abstract

The aim of this study was to investigate the effects of heat stress on the expression of the coxsackievirus and adenovirus receptor (CAR) in mouse skin keratinocytes. Twenty BALB/c mice were randomly divided into two groups: the sham heat (control) and scald groups. Skin specimens were obtained 6 h after the treatments. Changes in the expression of CAR in skin keratinocyte samples were detected by immunohistochemistry, quantitative polymerase chain reaction and western blotting. In an *in vitro* assay, mouse skin keratinocytes were cultured and randomly divided into two groups: the normal control and heat stress groups. Six hours subsequently, the changes in CAR expression in the two groups were estimated by flow cytometry to determine the differences between the two groups. Heat stress significantly increased the expression of CAR in the mouse skin keratinocytes (P<0.05). The upregulation of CAR in mouse keratinocytes in burn wounds may be beneficial for restoring healing in organisms.

## Introduction

Wound problems commonly occur in clinical practice; ∼l% of the global population suffers from persistent wound problems, and 5% of total medical costs are spent on wound treatments (1,2). Accurate wound treatment is required to solve this problem effectively, and such treatment relies on a better understanding of the wound healing mechanism. However, wound healing is a complex biological process. Different types of wounds have different healing processes and the mechanisms have not yet been fully elucidated. The exchange of signals among cells, transmembrane conductance and intracellular signal transduction are the potential physical mechanisms controlling the process. As a result, signal transduction mechanisms in wound healing have been the focus of the majority of studies. Witherden *et al* (3) observed an increased coxsackievirus and adenovirus receptor (CAR) expression near the keratinised cells in a mouse skin wound biopsy. It was demonstrated that CAR was involved in a cell-to-cell contact signalling pathway that resulted in an increase in the number of local cell growth factors and inflammatory mediators, thereby promoting wound healing. These results indicated that CAR functions as a signalling molecule in skin wound biopsies.

CAR is a 46 kDa type I transmembrane glycoprotein belonging to the immunoglobulin superfamily (4). In mice, CAR is mainly distributed in the heart, liver, brain, kidneys and lungs, with the highest expression level observed in the liver (5–7). Numerous studies have shown that CAR significantly functions as an important adhesion protein and molecule in viral infection (8–11) and the tumour development process (12–16). The correlation between CAR and wound healing was first described by Witherden *et al* (3). However, only the changes in CAR expression in the keratinocytes of skin wound biopsies have been observed; no other similar observations have been described for burn wounds, a different form of wound from a skin wound biopsy. To determine whether CAR exhibited similar expression changes in burn wounds, *in vitro* mouse and cellular experiments were performed in the present study. Such experiments were also conducted to observe the effects of the changes in CAR expression in thermally stimulated mouse skin keratinocytes. In addition, the function of CAR in the burn wound healing process was elucidated.

## Materials and methods

### Establishment of animal models

Twenty BALB/c mice (age, 6–8 weeks; 10 males/10 females; weight, 20–25 g; Academy of Military Medical Sciences, Beijing, China) were randomly divided into two groups. The mice remained awake while their backs were shaved. Following this, the depilated areas of one group (the scald group) were treated with hot water gauzes heated at 100°C for 1 to 3 sec. The other group (the sham heat or control group) was treated with humidity gauzes heated at room temperature. Six hours later, all the mice were sacrificed, and specimens of back skin were obtained for the subsequent experiment. This study was performed in strict accordance with the recommendations in the Guide for the Care and Use of Laboratory Animals of the National Institutes of Health. The animal use protocol was reviewed and approved by the Institutional Animal Care and Use Committee of the 309th Hospital of the Chinese PLA (Beijing, China).

### Keratinocyte culture and thermal stimulation model

The dorsal skin from BALB/c foetal mice (gestational age, 20 days) was washed with D-Hanks solution and cut into strips (0.3×1 cm). The skin was added to 1.25 U/ml dispase II solution (Roche, Basel Switzerland) and digested at 4°C for 24 h. Following this, the epidermis and dermis were separated, and the epidermis was cut in pieces, added to 1.25 *μ*/ml dispase II solution at 37°C and digested for 30 min. All of the tissue fragments were removed via a mesh filter, while the remaining cells were collected by centrifugation and cultured in Epilife medium (Invitrogen Life Technologies, Carlsbad, CA, USA). After 3 to 4 days, the fused cells were digested and passaged. The third generation of cells was used and divided into two groups for the subsequent experiments. The first group (the normal control group) was cultured at 37°C in 5% CO_2_ for 1 h; the other group (the heat stress group) was cultured at 42°C in 5% CO_2_ for 1 h. The two groups of cells were subsequently cultured under normal conditions for 6 h and then harvested.

### Immunohistochemistry

Following the dewaxing and hydration of the paraffin sections of the back skin samples, the antigens were retrieved by citric acid heating for 10 min. Endogenous catalases were blocked using freshly prepared 2% ddH_2_O at room temperature for 20 min, and non-specific binding sites were blocked by horse serum for 30 min. All the sections were incubated with a rabbit anti-mouse CAR antibody (Santa Cruz Biotechnology, Inc., Santa Cruz, CA, USA) at 4°C overnight and incubated with a secondary antibody (Santa Cruz Biotechnology, Inc.) at 37°C for 30 min. Avidin/Biotin Complex (ABC) reagents (Vector Laboratories, Burlingame, CA, USA) were added under the previous conditions. Diaminobenzidine (DAB) colour reactions were viewed under a microscope (5–10 min). The samples were dehydrated and sealed using graded ethanol, dimethylbenzene and resin. In the blank control group, the primary antibody was replaced with phosphate-buffered saline (PBS). Under the microscope, a granular appearance on the cell surface or a homogeneous brown reaction product was considered to be a positive result.

### Quantitative polymerase chain reaction (qPCR)

Total RNA was extracted from fresh skin specimens using RNeasy Plus Mini kit (Qiagen, Hilden, Germany) according to the manufacturer’s instructions. Total RNA (4 *μ*g) was subjected to reverse transcription using a transcriptor high fidelity cDNA synthesis kit (Roche). Following this, total RNA, random hexamer primer (2.0 *μ*l) and ddH_2_O were added to obtain a final volume of 11.4 *μ*l. The samples subsequently reacted at 65°C for 10 min and were placed immediately in an ice bath. Following this, 4.0 *μ*l 5X efficient reverse transcription buffer, 0.5 *μ*l RNase inhibitor, 2.0 *μ*l deoxynucleic acid mix, 1.0 *μ*l dithiothreitol (DTT) and 1.1 *μ*l efficient reverse transcription enzyme were added to obtain a final volume of 20 *μ*l. The following two reaction phases were conducted at 50°C for 30 min and 85°C for 10 min. The primer sequences were designed based on a GenBank (National Centre for Biotechnology Information, Bethesda, MD, USA) query: for CAR, forward primer, 5′-TACGAGTAACGATGTCAAGT-3′; reverse primer 5′-CCTGAAGGCTTAACAAGAAC-3′; for glyceraldehyde 3-phosphate dehydrogenase (GAPDH), forward primer 5′-TGAGTATGTCGTGGAGTC-3′; reverse primer 5′-CAATCTTGAGTGAGTTGTCAT-3′. The standard curve was then prepared. To detect the amplification efficiency of CAR and GAPDH by qPCR using RealmasterMix (SYBR-Green; Tiangen Biotechnology Co., Ltd., Beijing, China), the reverse transcription product cDNA was diluted five times by a gradient (5^0^, 5^1^, 5^2^, 5^3^ and 5^4^). The reaction system comprised 1 *μ*l cDNA, 9 *μ*l 5X PCR buffer, 0.4 *μ*l forward/reverse primer and 9.2 *μ*l ddH_2_O. The reaction conditions were as follows: one cycle of 95°C for 2 min; 94°C for 15 sec and 58°C for 10 sec; and 40 cycles of 68°C for 18 sec. The melting curve was analysed based on the following parameters: one cycle of 95°C for 1 min; one cycle of 55°C for 1 min; 57 cycles of 65–93°C, read board for 0.06 sec once a 0.5°C increase in temperature occurred, and read board for 0.05 sec once a 20°C increase in temperature occurred. Three duplicated wells and a negative control treatment with no template were set.

### Western blot analysis

Approximately 50 mg fresh skin specimens were homogenised with radio-immunoprecipitation assay (RIPA) lysis solution [10 mmol/l Tris HCl (pH 7.4), 150 mmol/l NaCl, 5 mmol/l EDTA, 1% Triton-X, 50 mmol/l NaF, 0.2 mmol/l Na_3_VO_4_, 1% sodium deoxycholate and complete mini EDTA-free protease inhibitor cocktail (Roche)]. The samples were completely homogenised for 30 min and allowed to crack. Following this, the samples were transferred into an EP tube and centrifuged at 8,000 × g for 20 min (4°C), prior to the supernatants being stored. The protein samples were electrophoresed by 15% sodium dodecyl sulphate-polyacrylamide gel electrophoresis (SDS-PAGE) and a transmembrane was formed at a constant voltage of 15 V for 1 h. The membranes were blocked by 5% skimmed milk at room temperature for 1.5 h and then incubated with anti-CAR (Santa Cruz Biotechnology, Inc.) and anti-GAPDH (Sigma, St. Louis, MO, USA) at 4°C overnight, as well as with goat anti-mouse secondary antibody (Earthox, LLC, San Francisco, CA, USA) at room temperature for 2 h. Enhanced chemiluminescence (ECL) fluorescence was determined using X-ray film in the dark. Grey-scale analysis was performed using Glyko Bandscan software (Glyko, Hayward, CA, USA).

### Flow cytometry

A Vybrant apoptosis kit (Invitrogen Life Technologies) was used to detect apoptosis. The cells were incubated overnight (4°C) with CAR rabbit anti-mouse antibody (Santa Cruz Biotechnology, Inc.) and then with goat anti-rabbit immunoglobulin (Ig) G-fluorescein isothiocyanate (FITC; Santa Cruz Biotechnology Inc.) secondary antibodies at room temperature for 2 h. Following this, the samples were washed with PBS to detect apoptosis.

### Statistical analysis

Experimental data were analysed using SPSS 12.0 statistical software (SPSS, Inc., Chicago, IL, USA). The CAR expression in two independent samples were compared using one way analysis of variance (ANOVA) and the Student’s t-test. P<0.05 was considered to indicate a statistically significant difference.

## Results

### Histological observation

**Figure 1.**
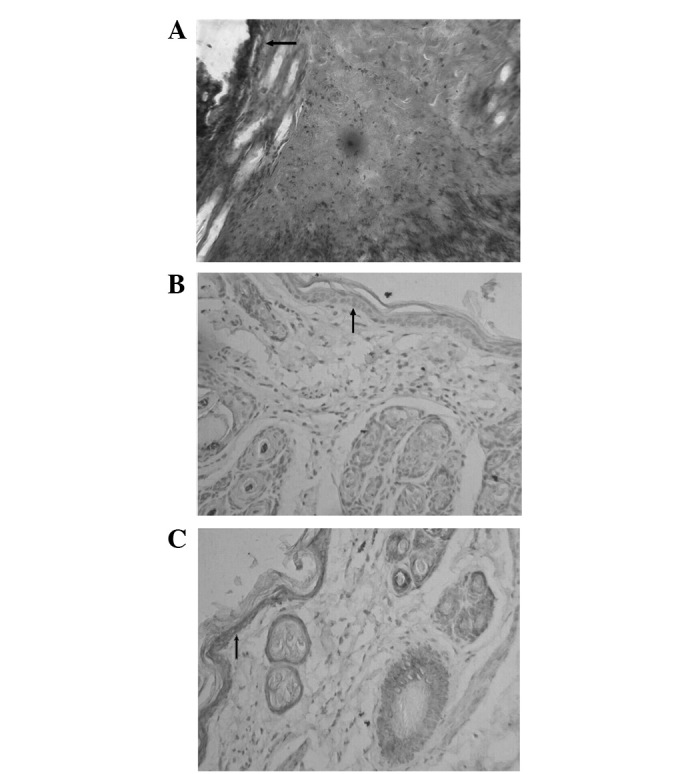
Histological observations. (A) Blank control; (B) epidermal keratinocytes of the normal mouse skin; (C) mouse skin epidermis following thermal stimulation. Immunostaining was used. The arrows indicate the skin epidermal keratinocytes where CAR may be expressed. Magnification, ×40.

No staining was observed in the blank control specimen (Fig. 1A). In the epidermal keratinocytes of the normal mouse skin (Fig. 1B), a mild positive staining was detected, suggesting that in normal circumstances mouse skin epidermal keratinocytes express a low level of CAR. Following thermal stimulation, the positive staining of the skin epidermis (Fig. 1C) was intensified. This result indicated that thermal stimulation may lead to the upregulation of CAR expression in mouse skin keratinocytes. CAR was also expressed in hair follicles, sweat glands, and epithelial cells. By contrast, no evident expression of CAR was observed in the skin fibroblasts.

### qPCR

**Figure 2.**
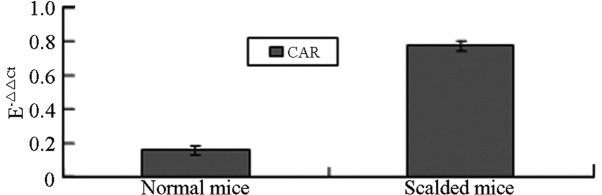
Effect of heat stress on the relative mRNA expression levels of the coxsackievirus and adenovirus receptor (CAR) in mouse keratinocytes.

In this study, the Pfaffl method (17) was used to evaluate the relative quantification of the CAR gene. The epidermal keratinocytes of the mouse skin were thermally stimulated and the results revealed that the CAR mRNA expression level of the thermally stimulated keratinocytes was significantly higher than that of the normal mouse keratinocytes (P<0.05; Table I and Fig. 2).

### Western blot

**Figure 3.**
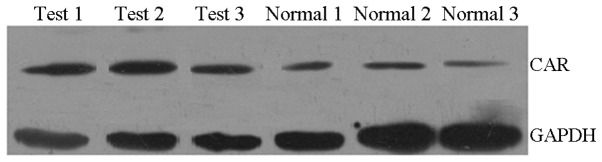
Relative expression of the coxsackievirus and adenovirus receptor (CAR) protein in heat-stressed (test) and normal mouse keratinocytes.

The grey-scale analysis was performed using Glyko Bandscan software and the grey-scale value of GAPDH was used as a reference. The ratio of the grey-scale value of the reference and the corresponding CAR value indicated the relative CAR expression. The results showed that there was a slight CAR protein expression in normal mouse skin keratinocytes and that this expression was increased in local skin keratinocytes following thermal stimulation (P<0.05; Table II and Fig. 3).

### Changes in apoptosis following heat stress

**Figure 4.**
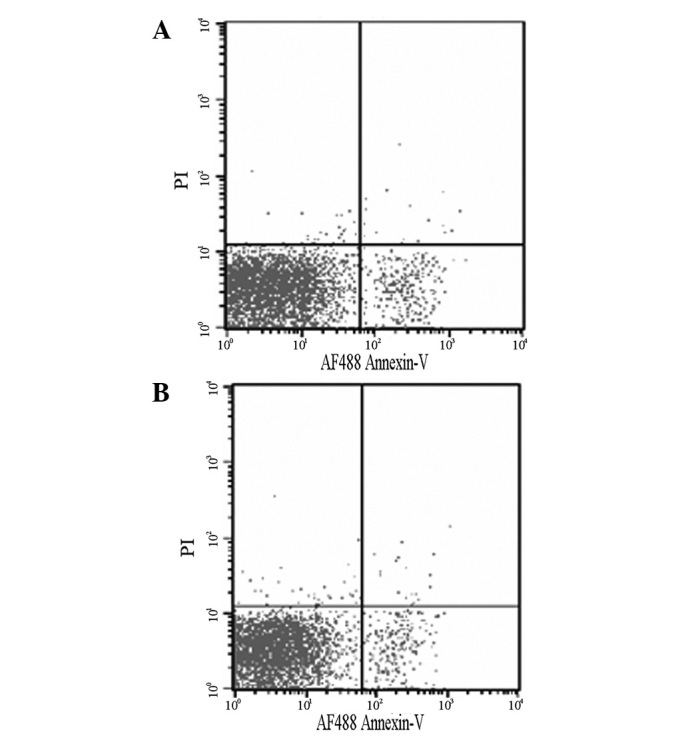
Detection of: (A) normal keratinocyte apoptosis; (B) keratinocyte apoptosis under heat stress.

Normal keratinocytes and thermally stimulated keratinocytes were subjected to apoptosis analysis. The apoptotic rates of the normal keratinocytes and thermally stimulated keratinocytes were 5.72±1.30 and 7.35±1.66%, respectively; however, these apoptotic rates were not significantly different (P>0.05; Fig. 4).

### CAR expression in keratinocytes following heat stress

**Figure 5.**
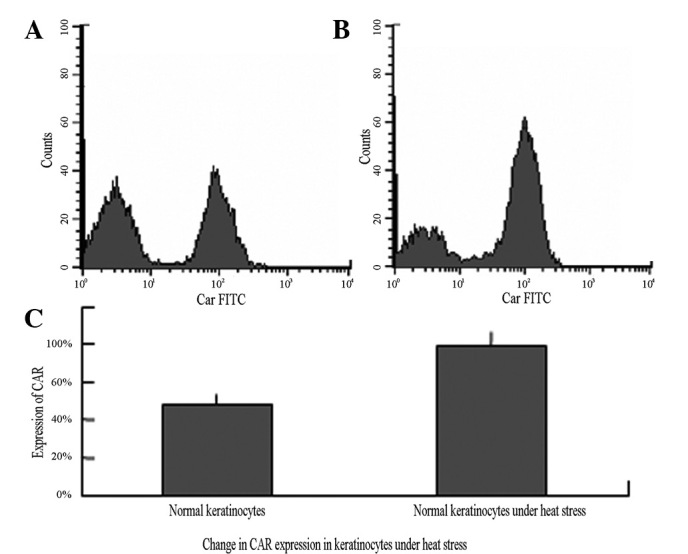
Expression of coxsackie-adenovirus receptor (CAR) in: (A) normal keratinocytes and (B) normal keratinocytes under heat stress. (C) CAR expression changes in keratinocytes under heat stress. FITC, fluorescein isothiocyanate.

In normally cultured keratinocytes, the expression rate of CAR was 48.36±5.07%; however, this rate significantly increased to 78.64±7.96% following the cells being subjected to heat stress (P<0.05; Table III and Fig. 5).

## Discussion

Wound healing occurs via different mechanisms. In contrast to skin wound biopsies, burn wounds do not undergo partial blood vessel rupture with bleeding and platelet aggregation at the vascular stump or the release of biologically active platelet-associated substances. Unlike burn wounds, skin wound biopsies exhibit few residual necrotic tissues, which may aggravate local inflammation. However, these wounds are acute; thus, their healing processes are similar (18). For keratinised epithelial cells, the increase in CAR expression in mouse skin wound biopsies was consistent with the upregulation in burn wounds. The experimental results confirmed this hypothesis, as the immunohistochemistry and western blot analyses of the skin tissues showed a significantly increased expression of CAR protein following thermal stimulation. qPCR further demonstrated that the expression of CAR mRNA in skin keratinocyte epithelial cells increased following heat stimulation, which suggests that the increase in the level of CAR expression may resulted from an enhancement of CAR gene transcription.

*In vitro* cell experiments were performed to determine whether the changes in CAR expression in keratinocytes following heat stress required signal exchanges with other cells. The primary keratinocytes underwent a strong thermal stimulation without the apoptotic rate being significantly affected; however, the CAR expression was significantly elevated. This result suggested that the changes in CAR expression following heat stress may be attributed to the self-stress response of cells, and that the thermally stimulated cells did not require signals from other cells to alter CAR expression.

The increased CAR expression level in the burn wounds of mice has a crucial function in wound healing. This result indicated that dendritic epidermal T cells (γδT cells, DETCs) are important in mouse skin wound healing (19); such cells may adjust to multiple factors of the process (20–23), particularly at the initiation of skin wound healing (23–27). However, mice with DETC dysfunction have an impaired wound healing response. A previous study (28) revealed that CAR in keratinocytes is able to combine with the junctional adhesion molecule-like protein (JAML) on the surface of DETCs and thereby enhance the activation level of the DETCs. This occurs via a co-stimulatory signal that passes via the phosphatidylinositol 3-kinase (PI3K)/Akt signalling pathway, thereby promoting the synthesis and secretion of interleukin (IL)-2, tumour necrosis factor (TNF) α, keratinocyte growth factor (KGF)-1 and interferon (IFN) γ, as well as DETC proliferation for wound healing.

At present, the effect of increased CAR expression in keratinocytes remains unclear. The increased CAR expression in different cells may be a result of different self-feedback systems. Okegawa *et al* (29) revealed that CAR was a potential growth inhibitory factor, and that increased CAR expression in bladder cancer cells induced the upregulation of p21. Subsequently, retinoblastoma (Rb) phosphorylation and accumulation led to cell cycle arrest at the G1 phase and/or apoptosis. Bagheri *et al* (30) also suggested that CAR was a growth inhibitory factor, due to the fact that bladder carcinoma cells with high CAR expression levels demonstrated a potent binding ability to oncolytic viruses, thus showing a good oncolytic therapeutic effect. Vindrieux *et al* (31) also described CAR as a potential type of unknown cytokine. In breast cancer patients, CAR expression is upregulated due to the effect of oestrogen, and this phenomenon is associated with cancer cell proliferation. Since the increased CAR expression in burn wound keratinocytes is a response to injury factors, self-feedback may be inhibitory. However, this requires further investigation.

Based on the positive effect of CAR on wound healing, Verdino *et al* (32) studied a monoclonal antibody HL4E10a, with a molecular structure similar to that of CAR, which was able to combine with JAMLs on DETCs. Although their binding sites were not identical, this antibody was able to effectively improve the level of activated DETCs. In the same study (32), the skin wounds of mice were treated with HL4E10 and a positive effect on wound healing was observed.

CAR is widely distributed in human tissues, and an 80% homology between human and mouse CAR nucleotide sequences has been identified (33). Similar to DETCs in mice, γδT cells are localised in the human skin (34,35). Therefore, there is a requirement for studies to be conducted to determine whether CAR and γδT cells in humans have the same functions as CAR and DETCs in mice. A better understanding of the function of CAR in wound healing may provide a new strategy for the treatment of wound problems.

## Figures and Tables

**Table I.** Relative quantitative expression of CAR gene.

Group	CAR Ct	GAPDH Ct	E-^ΔΔCt^
Normal mice	20.69±0.106	15.60±0.220	0.157±0.027
Scalded mice	19.89±0.018	16.98±0.048	0.773±0.029^a^

n=10 per group;

a P<0.05 compared with normal mice. CAR, coxsackievirus and adenovirus receptor; GADPH, glyceraldehyde 3-phosphate dehydrogenase.

**Table II.** Relative grey value of CAR protein.

Group	n	CAR/GAPDH
Normal mice	10	0.227±0.093
Scalded mice	10	0.891±0.144^a^

Results are presented as the mean ± standard deviation.

a P<0.05 compared with normal mice. CAR, coxsackie virus and adenovirus receptor; GADPH, glyceraldehyde 3-phosphate dehydrogenase.

**Table III.** Expression of CAR in keratinocytes detected using flow cytometry (mean ± SD)%.

Group	n	CAR expression
Normal keratinocytes	12	48.36±5.07
Keratinocytes after thermal stimulation	12	78.64±7.96^a^

a P<0.05 compared with normal keratinocytes. CAR, coxsackie-adenovirus receptor; SD, standard deviation.
